# Inflammatory Myofibroblastic Tumour of the Bladder in a Young Male: A Rare Case Report

**DOI:** 10.7759/cureus.83657

**Published:** 2025-05-07

**Authors:** Mohammad Hifzi Mohd Hashim, Suhaila Abdullah, Chin Yiun Lee, Syahril Anuar Salauddin, Hamid Hj Ghazali

**Affiliations:** 1 Department of Urology, Universiti Kebangsaan Malaysia Medical Center, Kuala Lumpur, MYS; 2 Department of Urology, Hospital Tengku Ampuan Afzan (HTAA), Kuantan, MYS; 3 Department of Pathology, Hospital Tengku Ampuan Afzan (HTAA), Kuantan, MYS

**Keywords:** anaplastic lymphoma kinase (alk), haematuria, inflammatory myofibroblastic tumour (imt), smooth muscle actin (sma), spindle cell neoplasm, turbt, urinary bladder tumour

## Abstract

Inflammatory myofibroblastic tumour (IMT) of the urinary bladder is a rare mesenchymal neoplasm characterised by spindle-shaped myofibroblastic cells and an accompanying inflammatory infiltrate. Although its aetiology remains unclear, histopathological and immunohistochemical analyses are essential for diagnosis. We report the case of an 18-year-old male who presented with sudden-onset, painless gross haematuria. Initial evaluation revealed severe anaemia, necessitating blood transfusion. Renal ultrasound demonstrated a large echogenic lesion within the bladder. Cystoscopy revealed a large, polypoidal tumour on the right lateral bladder wall with active bleeding. The tumour was completely resected via transurethral resection of bladder tumour (TURBT). Histopathological examination confirmed IMT, with immunohistochemical staining positive for smooth muscle actin (SMA) and anaplastic lymphoma kinase (ALK), supporting the diagnosis. The patient remained stable postoperatively, with no recurrence at three- and six-month follow-up. Bladder IMTs are uncommon, particularly in young males, and typically present with non-specific urinary symptoms, most notably haematuria. Differentiation from other spindle cell neoplasms, including urothelial carcinoma and sarcomas, is crucial for appropriate management. Complete surgical excision is typically curative, and ongoing surveillance with periodic cystoscopy is recommended due to the potential for recurrence. IMT of the bladder is a rare but important differential diagnosis for bladder tumours in young patients presenting with haematuria. Timely diagnosis and surgical intervention can lead to favourable outcomes, underscoring the importance of clinical awareness.

## Introduction

Inflammatory myofibroblastic tumours (IMTs) are rare neoplasms composed of spindle-shaped myofibroblastic cells with a variable inflammatory infiltrate [1]. These tumours can arise in various anatomical locations but are uncommon in the genitourinary tract, particularly in the bladder [2]. The exact aetiology remains uncertain, with proposed causes including reactive processes, infections, and genetic abnormalities [2]. Diagnosis relies on histopathological examination, with immunohistochemistry confirming the myofibroblastic nature of the spindle cells. Surgical resection remains the primary treatment, while adjuvant therapies such as chemotherapy or radiotherapy may be considered in aggressive or recurrent cases [1]. We present a case of a bladder IMT in an 18-year-old male with haematuria, highlighting diagnostic challenges, treatment strategies, and the importance of long-term follow-up. The diagnostic process can be challenging due to the tumour’s non-specific presentation and its potential to mimic more aggressive malignancies, often leading to misdiagnosis or delayed diagnosis.

## Case presentation

An 18-year-old male college student with no prior medical conditions, a non-smoker, and an active lifestyle presented to the ED with sudden-onset, painless gross haematuria. The haematuria was total, bright red, and profuse, and was associated with blood clots. He remained able to pass urine with clots without difficulty. He denied any recent trauma, vigorous physical activity, or sporting injury. There was no history of fever, back pain, lower urinary tract symptoms, or dysuria. He had no recent infections, was not on any medications or health supplements, and denied the use of recreational drugs. He was unmarried, had no sexual partner, and denied any high-risk sexual behaviour. There was no personal or family history of bleeding disorders or malignancy, and no recent travel history.

Despite ongoing haematuria, he initially delayed seeking medical attention but presented six hours later due to dizziness and palpitations, suggestive of symptomatic anaemia. On arrival, he appeared pale and tachycardic, with a heart rate of 126 beats per minute, blood pressure of 122/74 mmHg, and was afebrile at 36.8°C. Abdominal examination revealed a soft abdomen with a mildly palpable bladder. No abdominal wall ecchymosis or frank bruising was observed, helping to exclude underlying trauma or renal pathology such as spontaneous renal vessel rupture or perinephric haematoma. Genital examination was also unremarkable. A 3-way urinary catheter was inserted, and initial flushing removed blood clots, though active bleeding persisted. Continuous bladder irrigation was commenced, but haematuria remained profuse, and his haemoglobin level dropped to 5.0 g/dL. Perioperative blood investigations revealed preoperative leukocytosis, with normal coagulation and renal profiles (Table 1). He was resuscitated with intravenous crystalloid fluids and transfused with four units of packed red blood cells.

**Table 1:** Perioperative blood investigations demonstrating significant preoperative anaemia and leukocytosis, with normal coagulation and renal profiles. Postoperative improvements in haemoglobin and white cell counts were noted following transfusion and clinical stabilisation.

Blood Investigations Parameter	Pre-operative Value	Post-operative Day 1	Reference Range
Haemoglobin (g/dL)	5	9.2	13.0-17.0
White Cell Count (×10⁹/L)	22.6	11.4	4.0-11.0
Platelet Count (×10⁹/L)	300	233	150-400
Prothrombin Time (PT) (sec)	12.1	11.9	9.4-12.5
Activated Partial Thromboplastin Time (APTT) (sec)	30.1	29.5	28.1-39.7
International Normalised Ratio (INR)	1	1.1	0.8-1.2
Urea (mmol/L)	4.6	3.5	1.4-7.2
Creatinine (μmol/L)	82	71	59-104
eGFR (ml/min/1.73 m²)	122.6	131.4	>90

**Figure 1:**
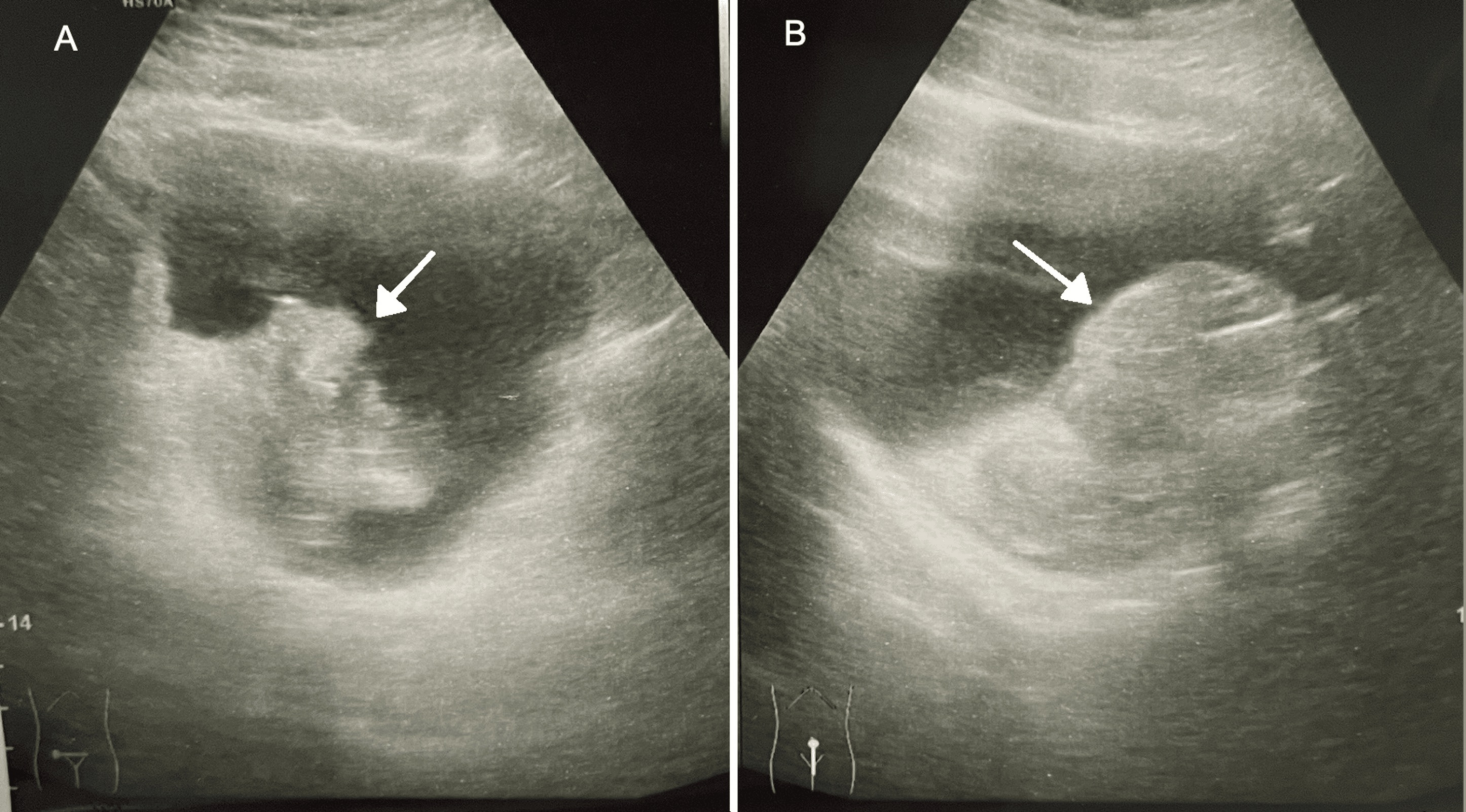
Ultrasound images of the bladder. The left panel (A) shows a transverse view, while the right panel (B) shows a longitudinal (sagittal) view. In both images, a white arrow indicates an echogenic lesion located at the dependent portion of the bladder base, which may represent either a blood clot or a tumour.

Renal USG demonstrated normal kidneys but revealed a large echogenic lesion at the dependent portion of the bladder, raising suspicion of a tumour or organised clot (Figure 1). In light of the ongoing bleeding and concerning sonographic findings, urgent cystoscopic evaluation and transurethral resection of bladder tumour (TURBT) were performed under general anaesthesia following informed consent.

Cystoscopy revealed a large, polypoidal, irregular tumour on the right lateral bladder wall with active bleeding (Video 1). The lesion was resected piecemeal using a bipolar loop, achieving complete excision and haemostasis. Tissue samples were sent for histopathological examination (HPE), labelled as bladder tumour and tumour base.

**Video 1:** Cystoscopic view demonstrating a bladder tumour on the right lateral wall, with active oozing of blood from its surface. Source: Provided by Dr. Mohammad Hifzi Mohd Hashim.

**Figure 2:**
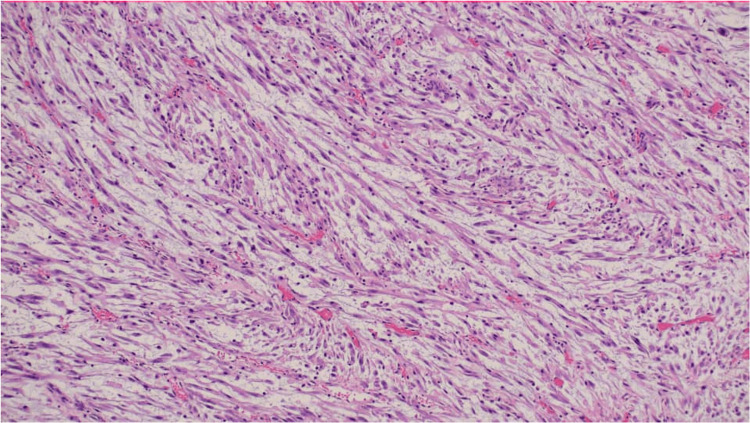
Proliferation of spindle cells arranged in poorly defined fascicles, with scattered inflammatory infiltrates. H&E stain, 100× magnification.

**Figure 3:**
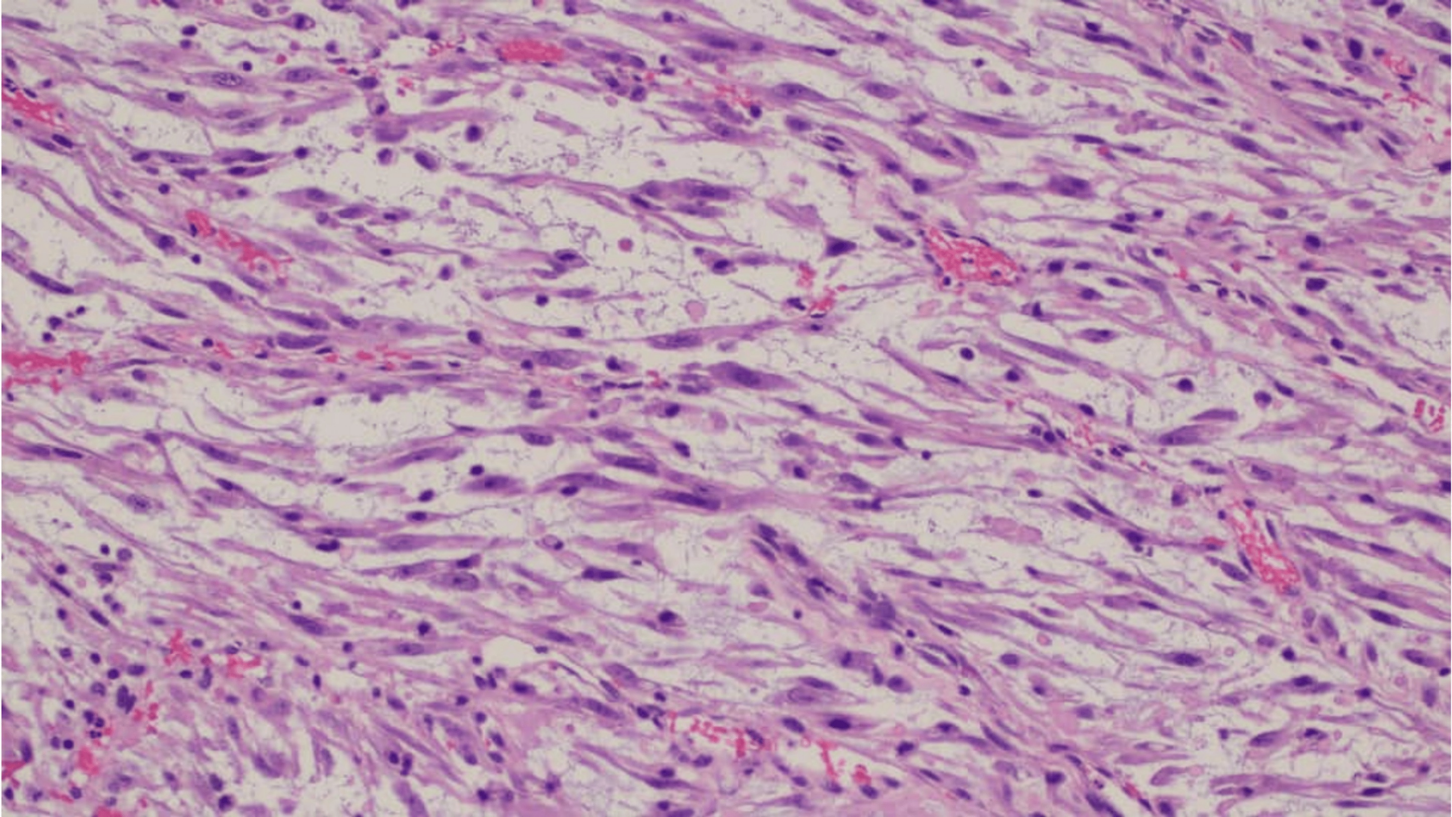
Spindle cells arranged in fascicles within a myxoid matrix, accompanied by blood vessels and inflammatory cells. H&E stain, 200× magnification.

**Figure 4:**
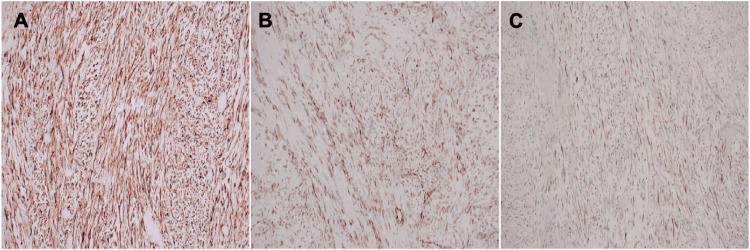
Immunohistochemical staining demonstrating (A) ALK positivity, (B) SMA positivity, and (C) desmin positivity. ALK: Anaplastic lymphoma kinase; SMA: Smooth muscle actin.

Microscopic examination showed a proliferation of spindle cells arranged in poorly defined fascicles within a myxoid stroma containing scattered small blood vessels (Figures 2-3). The tumour cells exhibited ovoid nuclei with mild-to-moderate pleomorphism and elongated cytoplasmic processes. A mild mixed inflammatory infiltrate was noted, along with occasional mitotic figures. The overlying urothelium was intact, without evidence of significant dysplasia, carcinoma in situ, or lymphovascular invasion. Immunohistochemical analysis revealed diffuse positivity for smooth muscle actin (SMA), anaplastic lymphoma kinase (ALK), desmin, and focal positivity for cytokeratin AE1/AE3 (Figure 4). The tumour was negative for S100 and CD34. These findings were consistent with a diagnosis of IMT of the bladder.

Postoperatively, the patient remained clinically stable with complete resolution of haematuria. Bladder irrigation was discontinued after 24 hours, and the urinary catheter was removed on postoperative day two, following which he voided normally. Postoperative improvement in haemoglobin and white cell count was observed following blood transfusion and clinical stabilisation (Table 1). He regained full functional capacity within a week and reported returning to his normal routine without difficulty. Final histopathology confirmed a benign IMT with clear margins. The case was discussed at a multidisciplinary team (MDT) meeting, and in view of the benign nature and complete resection, surveillance with cystoscopy alone was deemed sufficient; additional imaging was not considered necessary.

Follow-up cystoscopies at three and six months revealed healthy bladder mucosa with no evidence of recurrence or inflammation. The patient remained asymptomatic throughout the follow-up period, expressed satisfaction with his recovery, and demonstrated good compliance with surveillance. He agreed to continue with regular cystoscopic follow-up every six months for two years, followed by annual evaluations for five years.

## Discussion

IMTs are rare neoplasms composed of spindle-shaped myofibroblastic cells accompanied by an inflammatory infiltrate, including plasma cells, lymphocytes, and eosinophils [3]. Although they can arise in various anatomical locations, bladder involvement is uncommon, particularly among young patients. The aetiology remains uncertain, with potential contributing factors including immune dysregulation, infections, and genetic mutations [4].

Bladder IMTs are more frequently observed in young females and are exceedingly rare in paediatric populations [5]. A review by Teoh JY et al., which analysed 182 cases, reported a mean age of 38.9 years at diagnosis [6]. Clinical presentation is typically non-specific and may include haematuria, voiding symptoms, or lower abdominal discomfort [7]. Given the rarity of the tumour and its overlapping features with other lesions, definitive diagnosis depends on histopathological evaluation supported by immunohistochemical staining to distinguish IMTs from other spindle cell neoplasms. Prognosis is generally favourable, with surgical resection being the mainstay of treatment.

Diagnosis and histology

The differential diagnosis of bladder masses in young adults encompasses both benign and malignant entities. While urothelial carcinoma is more common in older populations, it can also occur in younger patients with predisposing risk factors such as smoking or occupational exposure [8]. Other considerations include inflammatory pseudotumours and sarcomas. In this case, the patient's young age, lack of risk factors, and acute presentation with haematuria prompted consideration of a benign or low-grade malignant lesion.

Histologically, IMTs are characterised by spindle-shaped cells arranged in fascicular or storiform patterns within a myxoid stroma, interspersed with a chronic inflammatory infiltrate [9]. Immunohistochemical staining is pivotal in differentiating IMTs from other spindle cell tumours. These tumours commonly express markers such as vimentin, SMA, and ALK [9]. ALK, a receptor tyrosine kinase, plays a key role in the tumourigenesis of IMTs, with gene rearrangements identified in a substantial proportion of cases. Detection of ALK expression via immunohistochemistry or fluorescence in situ hybridisation serves not only as a valuable diagnostic marker but also presents a potential therapeutic target [10].

Although ultrasonography is a useful initial imaging modality for evaluating pelvic and bladder abnormalities, its capacity to differentiate IMTs from other neoplastic and non-neoplastic conditions is limited. MRI, particularly with T2-weighted sequences, may suggest the presence of a fibroblastic or myofibroblastic lesion, though findings are not specific and must be correlated with histopathological results. Hence, definitive diagnosis still relies on tissue confirmation [11]. In this case, ultrasound was chosen as the first-line imaging modality due to its accessibility and speed in the emergency department, allowing prompt evaluation of the bladder and exclusion of upper urinary tract involvement in a haemodynamically unstable patient.

Treatment approaches

Surgical resection via TURBT or partial cystectomy remains the standard treatment modality [12]. Complete excision is essential to reduce the risk of recurrence, while adjuvant therapies may be considered in cases of residual or unresectable disease [13]. Given the rarity of bladder IMTs, optimal treatment strategies have yet to be firmly established [5]. In this case, TURBT was selected over partial cystectomy given the lesion’s endoscopic visibility, lack of deep invasion, and the urgent need to control active bleeding in a haemodynamically compromised patient. Close follow-up with regular cystoscopic surveillance is critical, particularly in the early years post-treatment, to detect potential recurrences [14].

Targeted therapies, especially ALK inhibitors, have demonstrated potential in the treatment of IMTs harbouring ALK gene rearrangements, particularly in metastatic or unresectable disease [15]. These agents function by selectively inhibiting the tyrosine kinase activity of the ALK fusion protein, which drives tumourigenesis through constitutive activation of downstream signalling pathways such as PI3K/AKT, RAS/ERK, and JAK/STAT. Although promising results have been reported in such advanced cases using crizotinib, a first-generation ALK inhibitor [15], the role of ALK inhibitors in the adjuvant setting for bladder IMTs remains undefined [10,16]. Treatment decisions should be individualised based on the patient’s overall health, tumour characteristics, and the risk-benefit profile of systemic therapy. Ongoing clinical trials and collaborative research are vital to developing more refined treatment strategies and improving outcomes in this rare tumour type.

Prognosis

Bladder IMTs generally follow a benign course, with surgical resection resulting in excellent local control and long-term survival [2,11]. While recurrence is rare, it may occur in cases involving larger tumours, infiltrative histological patterns, or ALK gene rearrangements [17]. IMTs are regarded as tumours of intermediate biological potential, given their low risk of distant metastasis but potential for local recurrence [18].

A multicentre study conducted over an 18-year period reported that patients with bladder IMTs had a largely benign disease trajectory, with a local recurrence rate of only 4% over a median follow-up of 43.4 months. Notably, no cases of distant metastasis were documented in this cohort [19].

Although the prognosis of bladder IMTs is generally favourable, diagnosis can be challenging due to their rarity and resemblance to malignant spindle cell tumours. Misdiagnoses have been reported, sometimes resulting in overtreatment, such as unnecessary radical cystectomy [20]. In our case, early cystoscopic evaluation and confirmatory histopathology allowed for accurate diagnosis and appropriate management. Continued surveillance is essential given the risk of local recurrence.

## Conclusions

This case underscores the importance of considering IMT as a differential diagnosis in young patients presenting with gross haematuria and a bladder mass. The diagnosis was confirmed through characteristic histopathological and immunohistochemical findings, notably ALK and SMA positivity. Prompt recognition and complete surgical excision via TURBT led to excellent postoperative recovery with no recurrence at six months. This case reinforces the value of early cystoscopic evaluation in atypical presentations and highlights the pivotal role of histopathology in guiding definitive management. While the prognosis is generally excellent, long-term cystoscopic surveillance remains essential due to the risk of recurrence. Further research is warranted to better define optimal treatment strategies and to improve understanding of the tumour’s biological behaviour.
